# HIV prevalence and behavioral and psychosocial factors among transgender women and cisgender men who have sex with men in 8 African countries: A cross-sectional analysis

**DOI:** 10.1371/journal.pmed.1002422

**Published:** 2017-11-07

**Authors:** Tonia Poteat, Benjamin Ackerman, Daouda Diouf, Nuha Ceesay, Tampose Mothopeng, Ky-Zerbo Odette, Seni Kouanda, Henri Gautier Ouedraogo, Anato Simplice, Abo Kouame, Zandile Mnisi, Gift Trapence, L. Leigh Ann van der Merwe, Vicente Jumbe, Stefan Baral

**Affiliations:** 1 Epidemiology Department, Johns Hopkins Bloomberg School of Public Health, Baltimore, Maryland, United States of America; 2 Biostatistics Department, Johns Hopkins Bloomberg School of Public Health, Baltimore, Maryland, United States of America; 3 Enda Sante, Dakar, Senegal; 4 Joint United Nations Programme on HIV and AIDS Country Office, Mbabane, Swaziland; 5 People’s Matrix Association, Maseru, Lesotho; 6 Programme d’Appui au Monde Associatif et Communautaire, Ouagadougou, Burkina Faso; 7 Institut de Recherche en Sciences de la Santé, Ouagadougou, Burkina Faso; 8 Institut Africain de Santé Publique, Ouagadougou, Burkina Faso; 9 ONG Arc-en-Ciel, Lomé, Togo; 10 Ministère de la Sante et de l’Hygiène Publique, Abidjan, Côte d’Ivoire; 11 Health Research Department, Strategic Information Division, Ministry of Health, Mbabane, Swaziland; 12 Centre for the Development of People, Lilongwe, Malawi; 13 Social, Health, and Empowerment Feminist Collective of Transgender Women of Africa, East London, South Africa; 14 Malawi College of Medicine, Blantyre, Malawi; Desmond Tutu HIV Centre, SOUTH AFRICA

## Abstract

**Introduction:**

Sub-Saharan Africa bears more than two-thirds of the worldwide burden of HIV; however, data among transgender women from the region are sparse. Transgender women across the world face significant vulnerability to HIV. This analysis aimed to assess HIV prevalence as well as psychosocial and behavioral drivers of HIV infection among transgender women compared with cisgender (non-transgender) men who have sex with men (cis-MSM) in 8 sub-Saharan African countries.

**Methods and findings:**

Respondent-driven sampling targeted cis-MSM for enrollment. Data collection took place at 14 sites across 8 countries: Burkina Faso (January–August 2013), Côte d’Ivoire (March 2015–February 2016), The Gambia (July–December 2011), Lesotho (February–September 2014), Malawi (July 2011–March 2012), Senegal (February–November 2015), Swaziland (August–December 2011), and Togo (January–June 2013). Surveys gathered information on sexual orientation, gender identity, stigma, mental health, sexual behavior, and HIV testing. Rapid tests for HIV were conducted. Data were merged, and mixed effects logistic regression models were used to estimate relationships between gender identity and HIV infection. Among 4,586 participants assigned male sex at birth, 937 (20%) identified as transgender or female, and 3,649 were cis-MSM. The mean age of study participants was approximately 24 years, with no difference between transgender participants and cis-MSM. Compared to cis-MSM participants, transgender women were more likely to experience family exclusion (odds ratio [OR] 1.75, 95% CI 1.42–2.16, *p <* 0.001), rape (OR 1.95, 95% CI 1.63–2.36, *p <* 0.001), and depressive symptoms (OR 1.30, 95% CI 1.12–1.52, *p <* 0.001). Transgender women were more likely to report condomless receptive anal sex in the prior 12 months (OR 2.44, 95% CI 2.05–2.90, *p <* 0.001) and to be currently living with HIV (OR 1.81, 95% CI 1.49–2.19, *p <* 0.001). Overall HIV prevalence was 25% (235/926) in transgender women and 14% (505/3,594) in cis-MSM. When adjusted for age, condomless receptive anal sex, depression, interpersonal stigma, law enforcement stigma, and violence, and the interaction of gender with condomless receptive anal sex, the odds of HIV infection for transgender women were 2.2 times greater than the odds for cis-MSM (95% CI 1.65–2.87, *p <* 0.001). Limitations of the study included sampling strategies tailored for cis-MSM and merging of datasets with non-identical survey instruments.

**Conclusions:**

In this study in sub-Saharan Africa, we found that HIV burden and stigma differed between transgender women and cis-MSM, indicating a need to address gender diversity within HIV research and programs.

## Abstract 2

In a cross-sectional analysis of data from African countries, Tonia Poteat and colleagues report on risk factors for infection and HIV prevalence in transgender women and men who have sex with men.

## Author summary

**Why was this study done?:**

- Sub-Saharan Africa includes countries with the most broadly generalized HIV epidemics.
- Transgender women have unmet HIV prevention and treatment needs around the world; however, study of their specific needs across sub-Saharan Africa has been limited.

**What did the researchers do and find?:**

- We merged HIV test results and survey data from studies initially focused on gay men and other men who have sex with men (MSM) in 8 countries in sub-Saharan Africa, then specifically assessed transgender women separately from cisgender MSM.
- We identified 937 transgender women among the total sample of 4,586 individuals.
- We found that transgender women were more likely than cisgender MSM to test positive for HIV as well as report experiences of stigma, depressive symptoms, and condomless sex.

**What do these findings mean?:**

- Gender identities are as complex across sub-Saharan Africa as they are in other regions.
- These data highlight the limitations of an essentialist gender binary framework for HIV prevention and treatment programs in sub-Saharan Africa.
- Advancing HIV prevention and treatment in sub-Saharan Africa necessitates specifically studying the appropriate content and implementation of programs that reach transgender women.

## Introduction

Sub-Saharan Africa bears more than 70% of the global burden of HIV [1]. Countries across the region have experienced broadly generalized HIV epidemics [2], and early studies focused on cisgender (non-transgender) heterosexual adults [3,4]. More recent epidemiological data have identified the uneven distribution of HIV risk, even within generalized epidemics [5,6]. As attention to the HIV-related needs of key affected populations such as men who have sex with men (MSM) and female sex workers grew [6,7], researchers began to explicitly assess gender diversity and notice higher HIV burdens among gender minorities [8,9]. However, very little research has addressed HIV among transgender women in sub-Saharan Africa [10–12], and HIV data from transgender women are often subsumed within MSM data [13].

Transgender women experience a disproportionate burden of HIV. The most recent global meta-analysis of HIV among transgender women examined laboratory-confirmed HIV data from 15 countries over the period of 2001–2011. The pooled HIV prevalence for transgender women was 19%, with this group having a 49-fold higher odds of infection compared with other reproductive age adults [14]. However, data were only available from countries with male-predominant epidemics, and no data were available from sub-Saharan Africa. A systematic review of the global HIV epidemiology among transgender populations was published in 2016, including research published between 2012 and 2015 [12]. Although data were available from more than 30 studies around the world, none were from sub-Saharan Africa. Of the 20 countries that reported HIV prevalence data on transgender people to the Joint United Nations Programme on HIV/AIDS (UNAIDS) in 2016, only 1, Democratic Republic of the Congo (DRC), was in sub-Saharan Africa [15]. DRC reported HIV prevalence of 7.9% among transgender people compared with 0.7% among adults 15–49 years old and 3.3% among MSM [16].

In 2016, Stahlman and colleagues published the first study of laboratory-confirmed HIV among transgender women in sub-Saharan Africa, with a focus on west Africa. They examined data from Togo, Burkina Faso, and Côte d’Ivoire [17] and found that 18% of 2,456 participants in studies targeting cisgender MSM (cis-MSM) identified as female or transgender. Compared with cis-MSM, transgender women in these studies reported greater levels of stigma and were more likely to be living with HIV. While sexual behavior stigma was not significantly associated with HIV prevalence, it was associated with condomless anal sex. This analysis was an important first step in filling the void of information on HIV in transgender women in sub-Saharan Africa. However, Togo, Burkina Faso, and Côte d’Ivoire have low-level epidemics with HIV prevalences of 2.4%, 0.8%, and 3.5%, respectively [2].

To date, no data have been published characterizing the HIV prevalence and associated risk factors among transgender women in eastern or southern Africa. To fill this gap, we analyzed data from HIV biobehavioral studies conducted between 2011 and 2016 across sub-Saharan Africa. The objectives of this analysis were to estimate HIV prevalence among transgender women, characterize psychosocial and behavioral risk factors, and distinguish HIV epidemiology among transgender women from that among cis-MSM.

## Methods

This secondary analysis of pooled data from multiple cross-sectional, biobehavioral studies did not use a formal written protocol or prospective analysis plan. The analyses were guided by the a priori research objectives described above. Details of the analysis plan history are provided in S1 Text.

### Study sample

Data for these analyses were collected in urban settings as part of larger cross-sectional studies initially tailored for MSM. Data collection took place from 2011 to 2016 at 14 sites across 8 countries: Bobo-Dioulasso and Ouagadougou in Burkina Faso (January–August 2013); Abidjan, Bouake, Gagnoa, and Yamoussoukro in Côte d’Ivoire (March 2015–February 2016); Banjul in The Gambia (July–December 2011); Maputsoe and Maseru in Lesotho (February–September 2014); Lilongwe in Malawi (July 2011–March 2012); Dakar in Senegal (February–November 2015); Mbabane in Swaziland (August–December 2011); and Kara and Lomé in Togo (January–June 2013).

Participants were recruited using respondent-driven sampling (RDS) [18], except in The Gambia where snowball sampling was used [19]. At sites that used RDS, recruitment was initiated using 3 to 6 seeds who were selected based on the recommendation of local community-based organizations. Seeds represented a range of characteristics in terms of age, education, socioeconomic status, and participation in lesbian, gay, bisexual, and transgender (LGBT) associations. In The Gambia, where there were no formal LGBT organizations, recruitment started with 10 highly motivated initial seeds who represented diversity in age and education levels and who were well-connected with members of the LGBT community.

Eligible participants were age 18 years and older in all countries except The Gambia, where enrollment included ages 16 years and older. Other eligibility criteria included being assigned male sex at birth and having had anal sex with a male partner in the prior 12 months. All participants provided verbal informed consent. Studies were approved by ethical review boards in each country as well as the Johns Hopkins Bloomberg School of Public Health Institutional Review Board.

### Data collection

To facilitate the privacy and safety of participants, data collection took place in study-specific facilities. During the study visit, interviewers administered a structured questionnaire including modules on demographics, gender and sexual identity, mental health, alcohol and substance use, sexual risk practices, and experiences of stigma. Interviews were conducted in English, French, or another local language (per participant choice) by trained peer or LGBT-friendly interviewers who were fluent in the languages used. Rapid HIV tests were conducted by trained staff following survey administration. HIV testing and counseling were done according to national guidelines in each country, including pre-test counseling and optional, but encouraged, post-test counseling. A serial rapid HIV testing algorithm was implemented using Determine HIV-1/2 (Alere, Japan) for screening and Uni-Gold HIV (Trinity Biotech Ireland) for confirmation of positive screening test results [20]. All participants who tested HIV-positive were referred for HIV care.

### Measures

Datasets from each country were merged, and all questions that were in at least 2 surveys were kept for analysis. Consistent with global standards [21], transgender women were defined as participants who were assigned male sex at birth and self-identified as transgender or female/woman. Three countries (Burkina Faso, Lesotho, and Togo) also included the option to identify as intersex; however, all intersex participants also identified as either male/men or female/woman/transgender. Therefore, gender was dichotomized as transgender women and cis-MSM. Demographic data included age in years and current employment status. Depression screening was conducted using an item that asked, “Have you ever felt sad or depressed in the last two weeks?” Suicidal ideation screening was conducted by “Have you ever felt like you wanted to end your life in the last two weeks?” Three questions assessed drug use: “Have you used needles to inject drugs in the last 12 months?” “If yes, have you used needles previously used by other people?” “Have you taken any drug that was not injected and not prescribed in the last 12 months?” Alcohol use was measured by “What is the average number of drinks you consume in one sitting?”

Sexual risk behaviors were assessed in multiple ways. In every country except Senegal and Côte d’Ivoire, participants were asked the number of male partners with whom they had anal sex in the prior 12 months. In Senegal and Côte d’Ivoire, respondents were asked about the number of anal sex acts or number of partners in the prior 30 days, but number of male anal sex partners could not be determined. Participants in all countries were asked whether a condom was used during the last time they had sex with a casual male partner and a regular male partner, and they were asked if they had condomless insertive and/or receptive anal sex with any of their reported male partners.

Scales used to measure access to condoms and lubricants varied by country. In 3 countries (Malawi, The Gambia, and Swaziland), participants were asked “What kind of access to condoms do you have when you need them?” with response options on a 4-point Likert scale where higher scores represent easier access. The same question was repeated replacing “condom” with “lubricant” in all countries except The Gambia, where lubricant access was not assessed. In 4 countries (Lesotho, Burkina Faso, Senegal, and Togo), participants were asked, “How difficult or easy is it for you to obtain condoms when you need them?” with response options on a 5-point Likert scale where higher scores represent easier access. The same question was repeated replacing “condom” with “lubricant.” Côte d’Ivoire data included the 5-point lubricant scale but no condom scale. Participants were also asked about history of sexually transmitted infection (STI) testing and diagnosis, and history of HIV testing and diagnosis.

Experiences of stigma based on sexual orientation/practice were measured using 13 questions representing 3 primary forms of stigma: enacted, anticipated, and perceived [22]. Questions asked about stigma from family, friends, and healthcare workers as well as in employment and education. Participants were asked about experiences of arrest, incarceration, torture, physical attacks, and rape. Finally, participants were asked about fear of walking in public spaces, fear of seeking health services, and denial of health services.

### Data analysis

Statistical analyses were conducted using R, an open-source software environment (R Foundation for Statistical Computing, Vienna, Austria) [23]. Means and proportions were calculated for participant characteristics and variables of interest. The number of respondents for each question (rather than the full dataset) was used as the denominator for calculating item proportions. Odds ratios (ORs) were used to compare results between transgender women and cis-MSM using univariate logistic regression with a random intercept to account for clustering by site.

A stigma index was created for item reduction using exploratory factor analysis (EFA) [24]. Varimax rotation was used due to minimal overlap in variance, and tetrachoric correlations were calculated to account for the binary nature of the items. Item reduction took place using an iterative process by which individual items were deleted if they failed to load on any factor or loaded equally on multiple factors; then, the EFA was conducted again on the lower number of items. Scree plots were used to determine the number of factors to extract. This process was repeated until each of the remaining items loaded (>0.5) on at least 1 but no more than 1 factor.

A mixed effects logistic regression model was built to estimate the odds of HIV infection for transgender women compared with cis-MSM [25]. A random intercept was added by site to account for clustering. The adjusted model included age as a potential confounder as well as stigma factor score, positive depression screen, and condomless receptive anal sex as potential mediators. We hypothesized that gender would modify the relationship between condomless receptive anal sex and HIV; therefore, an interaction term for gender and condomless receptive anal sex was included in the final model.

Because we combined data from multiple studies, no adjustments were made for RDS. The proportion of missing data was calculated for each variable in the model. Missing data comprised less than 5% of all variables, except condomless anal intercourse, which was missing for approximately 16% of participants, with no difference in proportion of missing data by gender. No data were imputed.

## Results

### Participant characteristics

As presented in Table 1, all participants were assigned male at birth; however, 937 (20%) identified as transgender or female while 3,649 were cis-MSM. The largest proportion of transgender participants were from Côte d’Ivoire (33%), followed by Senegal (21%), Swaziland (13%), Burkina Faso (12%), Malawi (8%), Lesotho (8%), Togo (6%), and The Gambia (<1%). Approximately 3% of transgender women and 12% of cis-MSM also identified as intersex in the 3 countries where this was assessed. The mean age of study participants was approximately 24 years, with no difference between transgender participants and cis-MSM. There was no difference in employment status by gender. The most frequently reported status for both groups was student (34% of transgender women and 37% of cis-MSM), followed by unemployed (19%).

**Table 1:** Participant characteristics (*N* = 4,586).

Characteristic	Transgender women (*N =* 937)	Cisgender MSM (*N =* 3,649)
**Age, mean (range)**	23.68 (16–56)	23.97 (16–62)
**Employment**		
Unemployed	181 (19.3)	689 (18.9)
Self-employed	148 (15.8)	578 (15.9)
Employed private/public sector	127 (13.6)	483 (13.3)
Student	319 (34.1)	1,339 (36.8)
Informal	82 (8.8)	211 (5.8)
Other	75 (8.0)	327 (9.0)
**Intersex**^1^		
Yes	8 (3.4)	203 (12.3)
No	224 (96.5)	1,446 (87.7)
**Country**		
Burkina Faso	109 (11.6)	563 (15.4)
Côte d’Ivoire	307 (32.8)	801 (22.0)
The Gambia	4 (0.4)	202 (5.5)
Lesotho	71 (7.6)	455 (12.5)
Malawi	75 (8.0)	263 (7.2)
Senegal	199 (21.2)	528 (14.5)
Swaziland	120 (12.8)	206 (5.6)
Togo	52 (5.5)	631 (17.3)

Data given as *N* (percent) unless otherwise indicated. Denominator is based on number of participants who were asked the question.

^1^Burkina Faso, Lesotho, and Togo were the only countries where respondents were asked about intersex status.

MSM, men who have sex with men.

### Behavioral and psychosocial factors

Prevalence of injection drug use was less than 2% for all participants. However, among injectors, injecting with a previously used needle was common (40% among transgender women and 25% among cis-MSM) and did not differ significantly by gender. Approximately 18% of both transgender women and cis-MSM had taken a drug that was not prescribed or injected in the prior 12 months, and both groups drank an average of approximately 3 drinks per sitting. Overall, there were no significant differences by gender in alcohol and substance use. Depressive symptoms and suicidal ideation were common among both transgender women (57% and 19%) and cis-MSM (47% and 16%). However, transgender women were significantly more likely to report feeling sad or depressed (OR 1.30, 95% CI 1.12–1.52, *p* < 0.001).

Of the 13 stigma items assessed, transgender participants were significantly more likely than cis-MSM to report experiencing 6 of them (Table 2). There were no differences by gender in loss of employment, denial of educational opportunities, having been tortured, having been arrested, having been to jail or prison, being afraid to seek health services, and having been denied health services. However, transgender participants were significantly more likely to report exclusion from family gatherings (OR 1.75, 95% CI 1.42–2.16, *p <* 0.001), discriminatory remarks by family (OR 1.57, CI 1.33–1.85, *p <* 0.001), rejection by friends (OR 1.47, CI 1.23–1.75, *p <* 0.001), being beaten up (OR 1.70, CI 1.43–2.02, *p <* 0.001), rape (OR 1.95, 95% CI 1.63–2.36, *p <* 0.001), and fear of walking in public spaces (OR 1.58, 95% CI 1.31–1.91, *p <* 0.001). For transgender participants, discriminatory remarks from family members was most common (35%), followed by being beaten up (33%) and being rejected by friends (30%). The EFA supported a 3-factor index (Table 3) that included interpersonal stigma (3 items), law enforcement stigma (2 items), and violence (2 items).

**Table 2:** Psychosocial factors among transgender women and cisgender MSM.

Survey item	Transgender women (*N =* 937)	Cisgender MSM (*N =* 3,649)	OR (95% CI)	*p*-Value
**Stigma**				
Have you ever felt excluded from family gatherings as a result of your sexual orientation or practice?	167 (18.3)	387 (11.1)	1.75 (1.42–2.16)	<0.001
Have you ever felt that family members have made discriminatory remarks or gossiped about you because of your sexual orientation or practice?	328 (35.1)	866 (23.8)	1.57 (1.33–1.85)	<0.001
Have you ever felt rejected by your friends as a result of your sexual orientation or practice?	279 (30.0)	790 (21.7)	1.47 (1.23–1.75)	<0.001
Have you ever lost employment as a result of your sexual orientation or practice?	16 (4.3)	55 (2.9)	1.52 (0.82–2.69)	0.162
Have you ever been denied educational opportunities, like access to school, as a result of your sexual orientation or practice?	16 (3.7)	73 (3.2)	1.07 (0.58–1.87)	0.819
Have you ever been tortured?	74 (17.2)	244 (10.6)	1.00 (0.72–1.37)	0.990
Have you ever been arrested?	72 (7.9)	277 (7.7)	0.95 (0.71–1.26)	0.726
Have you ever felt scared to walk around in public spaces as a result of your sexual orientation or practice?	229 (24.4)	527 (14.5)	1.58 (1.31–1.91)	<0.001
Have you ever been beaten up/physically attacked?	311 (33.2)	791 (21.7)	1.70 (1.43–2.02)	<0.001
Have you ever been raped?	257 (27.6)	520 (14.3)	1.95 (1.63–2.36)	<0.001
Have you ever been to jail or prison?	48 (5.2)	167 (4.6)	0.91 (0.64–1.28)	0.585
Have you ever felt afraid to seek healthcare services as a result of your sexual orientation or practice?	250 (26.7)	762 (20.1)	1.13 (0.95–1.36)	0.169
Have you ever been denied health services as a result of your sexual orientation or practice?	14 (2.2)	46 (1.6)	1.24 (0.64–2.27)	0.501
**Mental health**				
Have you ever felt sad or depressed in the last two weeks?	536 (57.3)	1624 (44.7)	1.30 (1.12–1.52)	<0.001
Have you ever felt like you wanted to end your life in the last two weeks?	178 (19.1)	562 (15.5)	1.06 (0.86–1.29)	0.587
**Alcohol and substance use**				
Have you used needles to inject drugs in the last 12 months?	5 (1.1)	36 (1.5)	0.42 (0.13–1.07)	0.083
If yes, have you used needles previously used by other people?	2 (40.0)	9 (25.0)	1.01 (0.14–4.69)	0.986
Have you taken any drug that was not injected and not prescribed in the last 12 months?	166 (17.9)	657 (18.4)	0.83 (0.68–1.02)	0.076
What is the average number of drinks you consume in one sitting? (mean)	2.91	2.83	—	0.210

Data are given as *N* (percent) answering “yes,” unless otherwise indicated. Percent calculated based on number who responded to the question. Rows with statistically significant differences between transgender women and cis-MSM are shaded yellow. ORs adjusted by site.

MSM, men who have sex with men; OR, odds ratio.

**Table 3:** Stigma index item factor loadings.

Item or measure	Factor 1	Factor 2	Factor 3
**Interpersonal stigma**			
Excluded from family gatherings	**0.91**	0.17	0.17
Stigma by family	**0.87**	0.03	0.32
Rejected by friends	**0.68**	0.07	0.02
**Law enforcement stigma**			
Arrested	0.01	**0.77**	0.39
Jailed	0.14	**0.85**	0.05
**Violence**			
Beat up	0.17	0.02	**0.58**
Raped	0.07	0.12	**0.52**
**Measure**			
SS loadings	2.099	1.360	0.895
Proportion of variance	0.300	0.194	0.128
Cumulative variance	0.300	0.494	0.622

Item loadings are bold in the column of their associated factor.

SS, sum of squared loadings.

### Sexual risk behavior

Table 4 presents comparative data on sexual behavior, condom and lubricant access, and HIV and STI history. Transgender women had a higher median number of male anal sex partners than cis-MSM (3 versus 2, *p =* 0.004) and were less likely to have used a condom the last time they had sex with a regular partner (OR 0.8, CI 0.67–0.96, *p =* 0.16). However, there was no statistically significant difference by gender in condom use at last sex with a casual partner. Transgender women had more than twice the odds of reporting condomless receptive anal sex with male partners compared to cis-MSM (OR 2.44, 95% CI 2.05–2.90, *p <* 0.001). Both groups found it easier to access condoms than lubricants. Transgender women reported greater access to condoms when they needed them than cis-MSM (3.4 versus 3.1 on a 4-point Likert scale, *p* < 0.001), and they reported easier access to lubricant when they needed it (3.6 versus 3.0 on a 5-point Likert scale, *p* < 0.001). Transgender women were less likely to have been tested for STIs in the prior 12 months (OR 0.81, 95% CI 0.65–0.99, *p =* 0.046) but had no significant difference from cis-MSM in STI diagnosis (8% versus 7%). There was no difference by gender in history of ever having had an HIV test (78% versus 75%) or having been tested in the prior 12 months (51% versus 44%).

**Table 4:** Sexual risk and HIV/STIs among transgender women and cisgender MSM.

Survey item	Transgender women (*N =* 937)	Cisgender MSM (*N =* 3,649)	OR (95% CI)	*p*-Value
**Sexual behavior**				
How many male partners have you had anal sex with in the last 12 months?^1^ median (IQR)	3 (2–5)	2 (1–4)	—	0.004
Of those male partners, have you had any unprotected insertive anal sex?^2^	180 (22.2)	1,184 (37.7)	0.52 (0.43–0.63)	<0.001
Of those male partners, have you had any unprotected receptive anal sex?^2^	331 (40.4)	757 (24.3)	2.44 (2.05–2.90)	<0.001
Was a condom used the last time you had sex with a regular partner?^2^	582 (70.1)	2,419 (75.2)	0.80 (0.67–0.96)	0.016
Was a condom used the last time you had sex with a casual partner?	485 (76.7)	1,776 (78.8)	1.03 (0.82–1.30)	0.790
**Condom/lubricant access, mean score**				
What kind of access to condoms do you have when you need them? (4-point scale: higher score is easier access)^3^	3.39	3.08	—	<0.001
How difficult or easy is it for you to obtain condoms when you need them? (5-point scale: higher score is easier access)^4^	4.32	4.31	—	0.915
What kind of access to lubricant do you have when you need it? (4-point scale: higher score is easier access)^5^	2.42	2.55	—	0.224
How difficult or easy is it for you to obtain lubricants when you need them? (5-point scale: higher score is easier access)^6^	3.64	3.01	—	<0.001
**Sexually transmitted infections**				
Have you been tested for STIs in the last 12 months?	138 (14.9)	701 (19.4)	0.81 (0.65–0.99)	0.046
Have you been told you have an STI in the last 12 months?	75 (8.2)	238 (6.7)	1.12 (0.84–1.49)	0.11
**HIV**				
Have you ever been tested for HIV?	632 (77.9)	2,409 (74.6)	1.17 (0.97–1.42)	0.104
Have you been tested for HIV in the last 12 months?	373 (50.5)	1,035 (44.3)	1.17 (0.99–1.39)	0.070
Have you been told by a doctor that you have HIV?	84 (11.1)	124 (4.4)	2.78 (2.03–3.81)	<0.001
Results from HIV test (% positive)	235 (25.4)	505 (14.1)	1.81 (1.49–2.19)	<0.001

Data are given as *N* (percent) answering “yes,” unless otherwise indicated. Percent calculated based on number who responded to the question. Rows with statistically significant differences between transgender women and cis-MSM are shaded yellow. ORs adjusted by site.

^1^Excluding Côte d’Ivoire and Senegal, where number of male anal sexual partners could not be ascertained.

^2^Denominator is the entire dataset, including both those who reported insertive and those who reported receptive anal sex.

^3^Asked in Malawi, The Gambia, and Swaziland.

^4^Asked in Lesotho, Burkina Faso, Senegal, and Togo.

^5^Asked in Malawi and Swaziland.

^6^Asked in Lesotho, Burkina Faso, Côte d’Ivoire, Senegal, and Togo.

CI, confidence interval; IQR, interquartile range; MSM, men who have sex with men; OR, odds ratio; STI, sexually transmitted infection.

### HIV status

Compared with cis-MSM, transgender women were more likely to have been told they had HIV in the past (OR 2.78, 95% CI 2.03–3.81, *p <* 0.001) and to have tested positive on the rapid HIV test conducted during the study (25% versus 14%; OR 1.81, 95% CI 1.49–2.19, *p <* 0.001) (Table 4). As expected, HIV prevalence varied widely by country (Table 5). In 4 of the 8 countries (Côte d’Ivoire, Lesotho, Senegal, and Togo), transgender women had significantly higher HIV prevalence than cis-MSM, and, in the remainder, there was no statistically significant difference by gender. HIV prevalence was highest in Lesotho, at 59% among transgender women compared with 29% among cis-MSM (OR 3.63, 95% CI 2.17–6.17, *p <* 0.001).

**Table 5:** HIV prevalence by country.

Country	HIV prevalence, percent (*n/N*)		OR (95% CI)	*p*-Value
	Transgender women	Cisgender MSM		
Burkina Faso	3 (3/108)	3 (18/552)	0.85 (0.20–2.56)	0.794
**Côte d’Ivoire**	**26 (76/298)**	**8 (61/766)**	**2.96 (2.00–4.38)**	**<0.001**
**The Gambia**	**50 (2/4)**	**9 (18/200)**	**10.11 (1.16–88.5)**	**0.025**
**Lesotho**	**59 (42/71)**	**29 (130/455)**	**3.63 (2.17–6.17)**	**<0.001**
Malawi	16 (12/75)	15 (40/263)	1.06 (0.51–2.09)	0.867
**Senegal**	**39 (74/199)**	**28 (147/528)**	**1.53 (1.08. 2.16)**	**0.015**
Swaziland	14 (17/120)	19 (38/204)	0.72 (0.38–1.32)	0.303
Togo	18 (9/51)	8 (53/626)	1.21 (0.52–2.58)	0.630
**Total**	**25 (235/926)**	**14 (505/3,594)**	**1.81 (1.49–2.19)**	**<0.001**

Percentages based on all available HIV test results, excluding missing values. ORs adjusted for site within each country. Rows with statistically significant differences between transgender women and cisgender MSM are in bold.

MSM, men who have sex with men; OR, odds ratio.

In multivariable regression modeling including age, stigma experiences, condomless receptive anal sex, positive depression screening, and the interaction between gender and condomless receptive anal sex, transgender women had twice the odds of testing positive for HIV (OR 2.17, 95% CI 1.65–2.97) compared with cis-MSM (Table 6). The variables age, positive depression screen, condomless receptive anal sex, law enforcement stigma, violence, and gender by condomless receptive anal sex were significantly associated with HIV in the adjusted model, while interpersonal stigma was not statistically significant.

**Table 6:** Multivariable logistic regression of odds of HIV infection.

Variable^1^	OR (95% CI)	*p*-Value
**Gender (transgender women versus cis-MSM)**	2.17 (1.65–2.87)	<0.001
**Age (1-year intervals)**	1.10 (1.08–1.12)	<0.001
**Condomless receptive anal sex**	2.12 (1.66–2.72)	<0.001
**Depression screen**	1.48 (1.21–1.81)	0.001
Interpersonal stigma	1.04 (0.93–1.16)	0.507
**Law enforcement stigma**	1.13 (1.02–1.24)	0.016
**Violence**	1.20 (1.07–1.35)	0.002
**Gender × condomless receptive anal sex**	2.14 (1.56–2.92)	<0.001

^1^Variables in bold are statistically significant.

cis-MSM, cisgender men who have sex with men; OR, odds ratio.

## Discussion

In this analysis of data from MSM-tailored studies in 8 countries across sub-Saharan Africa, 1 out of every 5 participants (*n =* 937) identified as transgender or as a woman. Transgender women were more likely to report stigma, depressive symptoms, condomless receptive anal sex, and receipt of an HIV test within the prior 12 months compared with cis-MSM. HIV prevalence was 25% among transgender women and 14% among cis-MSM. In adjusted regression modeling, transgender women demonstrated a 2-fold higher odds of HIV infection than cis-MSM, with significant effect modification by the interaction between gender and condomless receptive anal intercourse.

These data contribute important nuance to understanding the HIV epidemic in sub-Saharan Africa, where essentialist notions of binary gender have been perpetuated in HIV research and surveillance [10]. Documentation of precolonial gender and sexual diversity in countries across the African continent has existed for more than 100 years [26]. In recent years, transgender Africans have demanded increased visibility—publishing narratives of their experience [27], establishing and leading advocacy organizations [28–30], and participating in feature stories [31] and documentaries about their lives [32]. By assessing gender identity separately from biological sex assigned at birth, HIV researchers and systems can make transgender participants visible within the data. These findings highlight the importance of collecting data on gender identity and disaggregating the data, even in countries with generalized HIV epidemics. Accurately characterizing HIV epidemiology by gender is essential to the development of appropriate strategies that reach key populations at highest risk for HIV infection [33].

Overall, 1 in 4 transgender women tested positive for HIV infection across these studies. While HIV prevalence varied greatly by country, wherever there was a statistically significant difference between transgender women and cis-MSM, transgender women had a higher HIV prevalence. Even in Lesotho, which has a broadly generalized epidemic and national HIV prevalence of 25% [34], the HIV prevalence among transgender women was 59%—representing a 3.6-fold increased odds of infection compared with cis-MSM. Such stark disparities are consistent with data from other regions of the world [12,14] and highlight the urgent need to address HIV prevention and care for transgender women across sub-Saharan Africa, where their needs have long been ignored [10]. The high HIV prevalence among such a young sample, with an average age of 24 years, reinforces the importance of intervening early to prevent HIV in this population.

Transgender women in this study were more likely than cis-MSM to report condom use with their last regular partner and easier access to condoms and lubricants. However, they also reported a greater number of sexual partners and were more likely to report condomless receptive anal sex than cis-MSM. Receptive condomless anal intercourse with a serodiscordant and viremic partner has a relatively high per-act HIV acquisition probability [35,36]; therefore, one would expect receptive condomless anal sex with a high number of partners to be associated with HIV infection. However, the significant effect modification by gender on the relationship between condomless receptive anal sex and HIV suggests a more complex relationship. Transgender women were more likely than cis-MSM to have been recently tested for HIV; therefore, one possibility is that those individuals who recently received an HIV diagnosis and post-test counseling were more likely to use condoms. Alternatively, these data may suggest the need to look beyond behavioral risks and examine psychosocial and structural drivers of HIV infection.

Fewer study participants reported substance use than in prior research among transgender women in places like Brazil [37], Thailand [38], and the United States [39] or among MSM in southern Africa [40–42]. However, depression, suicidal ideation, and experiences of stigma were common. Transgender participants were more likely to report experiences of stigma and poor mental health than cis-MSM. Our findings that law enforcement stigma, violence, and depression were significantly associated with HIV are consistent with syndemic theories that structural and psychosocial factors are important synergistic drivers of HIV in gender and sexual minority populations [12].

The UNAIDS 2016–2021 Strategy aims for 90% of people living with HIV to know their status, 90% of people with diagnosed HIV infection to receive antiretroviral treatment (ART), and 90% of people taking ART to be virally suppressed by 2020 [43]. The strategy also sets a target for 90% of key populations, including transgender people, to have access to HIV combination prevention services [44]. Combination prevention includes traditional interventions such as condoms and lubricants as well as newer strategies such as pre-exposure prophylaxis (PrEP) and suppressive ART [45]. HIV self-testing [46], mobile technologies [47], and long-acting antiretroviral agents [48] are being explored as strategies to increase access to effective HIV prevention and care. However, these strategies are unlikely to be effective for transgender women without an enabling environment that reduces stigma, addresses the associated mental health burden [49], and provides culturally and biomedically appropriate HIV services [50].

Emerging data suggest that provision of gender-affirming care may reduce HIV-related risk [51] and improve engagement in the HIV prevention and care continuum [52,53] for transgender women. Testing interventions among transgender women in sub-Saharan Africa and ensuring that the results are utilized will be critical to ensuring that transgender women in the region are not left behind as countries implement their national HIV plans. In particular, as many countries scale up access to PrEP, important questions of acceptability [54], adherence [55], and potential drug–drug interactions with feminizing hormone therapies [56] must be addressed to facilitate engagement of transgender women.

This study marks a significant step forward in raising the visibility of the HIV epidemic among transgender women in sub-Saharan Africa. However, the findings may not be generalizable to other transgender women in the region due to the use of sampling strategies tailored for cis-MSM. While studies in other regions that specifically recruited transgender women consistently demonstrated a high prevalence of HIV in this population [12], emerging transgender-specific data support associations between access to gender affirmation and HIV risk [51], and it remains a testable hypothesis that transgender women with early access to legal, medical, social, and psychological gender affirmation may be less likely to participate in cis-MSM social networks and also less likely to experience psychosocial drivers of HIV infection.

These data are limited by their cross-sectional nature, which precludes causal inference. The use of face-to-face interviews for data collection may have led to social desirability bias, such as underreporting of HIV risk behaviors. While merging datasets across multiple sites allowed for greater statistical power in the analyses, potentially relevant variables were not included if they only existed in 1 dataset. Finally, interpretation of experiences of stigma was limited because some sites asked participants to attribute their experiences specifically to sexual orientation and practices, while others did not.

### Conclusions

Taken together, these data reinforce that gender identity is as complex in sub-Saharan Africa as in other regions, highlighting the need to collect and disaggregate data that distinguish assigned sex at birth from current gender identity. Most importantly, the data provide insights into where interventions are needed to mount an effective HIV response that considers the unique risks and vulnerabilities of transgender women in sub-Saharan Africa. Healthcare providers may use these data, particularly the high prevalence of stigma and violence, to motivate gender-affirming [51], trauma-informed care practices [57,58]. Globally relevant documents are available to guide healthcare providers in implementing HIV services for transgender individuals [59,60].

Appropriately powered studies specifically tailored for transgender women in sub-Saharan Africa would facilitate the robust analyses needed to fill gaps in knowledge of diverse transgender populations and to identify which transgender populations may be most vulnerable to HIV. Knowing that stigma drives both depression [61] and vulnerability to HIV infection, funders and national governments should invest in routine stigma surveillance [62] and the scale-up of effective transgender-inclusive stigma-reduction interventions [63,64] as key components of their HIV strategies. Transgender populations are emerging across sub-Saharan Africa: the data presented here suggest the need to understand the HIV epidemiology and specific HIV prevention and treatment needs of transgender populations in this region.

## Supporting information

S1 Text Analysis plan history. (DOCX)

S2 Text STROBE statement. (DOC)

## Abbreviations

ART: antiretroviral treatment
cis-MSM: cisgender men who have sex with men
EFA: exploratory factor analysis
LGBT: lesbian, gay, bisexual, and transgender
MSM: men who have sex with men
OR: odds ratio
PrEP: pre-exposure prophylaxis
RDS: respondent-driven sampling
STI: sexually transmitted infection
UNAIDS: Joint United Nations Programme on HIV/AIDS
