# Supplementary material for: HIV prevalence and behavioral and psychosocial factors among transgender women and cisgender men who have sex with men in 8 African countries: A cross-sectional analysis
Source: PLoS Med. 2017 Nov 7;14(11):e1002422. doi: 10.1371/journal.pmed.1002422 (PMC5675306; doi:10.1371/journal.pmed.1002422)
Supplement: S1 Text — (DOCX) [file pmed.1002422.s001.docx]

**S1 Text. Analysis history for “HIV prevalence and behavioral and psychosocial factors among transgender women and cisgender men who have sex with men in 8 African countries: a cross-sectional analysis”**

We did not publish a detailed analysis plan for this study. The analysis of data is described in the study methods in the main manuscript. Details on the history of this study are below:

1. Original data were collected from July 2011 - February 2016, including questionnaire and HIV testing data. Individual data sets for each country were cleaned and stored on a private server at Johns Hopkins University School of Public Health.
2. The study was motivated by the primary research question “Do transgender women in sub-Saharan Africa differ in HIV prevalence and risk factors from cisgender MSM?” This study question was defined prior to the analysis of any data.
3. A doctoral student was engaged to begin the process of merging and cleaning the data sets in October 2015, prior to the analysis of any data. The process of combining data sets to extract maximum information included decisions described in the main manuscript about operationalization of gender and merging of variables whose wording varied between countries. A final clean data set that included all countries was ready for analysis in June 2016.
4. At this point, specific research questions/hypotheses were generated. They included: 1) What proportion of recruited participants identified as female or transgender? 2) Is there any difference in HIV prevalence between trans women and cis men who have sex with men (cis-MSM)? and 3) What are the psychosocial factors that may contribute to any differences in HIV risk between these two groups? We hypothesized that transgender women would have significantly higher odds of HIV infection compared with cis-MSM, and that variables related to stigma and mental health would be associated with increased risk of HIV, in addition to traditional risk factors like condomless receptive anal sex.
5. From July 2016 – January 2017, exploratory analyses were conducted to better understand the study population and to determine the proportion of recruited participants who identified as female or transgender. Descriptive statistics of each variable were calculated, and compared across gender groups using t-tests. In order to reduce the dimensionality of the stigma variables, EFA was implemented to reduce the 13 individual stigma items to 3 factors for inclusion in the multivariable model. Finally, to assess the relationship between gender and HIV, a mixed effect logistic regression model was constructed that included a random intercept to account for clustering by site. Variables were included in the model based on *a priori* expectations that age was a potential confounder and that depression, stigma, and condomless receptive anal sex were potential mediators.
6. After peer-review from PLOS Medicine, the following new analyses were conducted: (a) An interaction term was created for gender by condomless receptive anal sex to test for effect modification in the multivariable model. (b) Mean number of sexual partners was replaced with the median and interquartile range. (c) We calculated percent missing data for key variables and compared missing data by question and between transgender women and cis-MSM. (d) We estimated effect sizes (odds ratios) for each relevant variable in Tables 2, 4, and 5.
