# Supplementary material for: HIV prevalence and behavioral and psychosocial factors among transgender women and cisgender men who have sex with men in 8 African countries: A cross-sectional analysis
Source: PLoS Med. 2017 Nov 7;14(11):e1002422. doi: 10.1371/journal.pmed.1002422 (PMC5675306; doi:10.1371/journal.pmed.1002422)
Supplement: S2 Text — (DOC) [file pmed.1002422.s002.doc]

**S2 Text. STROBE Statement for HIV prevalence and behavioral and psychosocial factors among transgender women and cisgender men who have sex with men in 8 African countries: a cross-sectional analysis**

|  | Item No | Recommendation | Paragraph Number by Section and Excerpted Text |
| --- | --- | --- | --- |
| **Title and abstract** | 1 | (*a*) Indicate the study’s design with a commonly used term in the title or the abstract | Title page: “HIV prevalence and behavioral and psychosocial factors among transgender women and cisgender men who have sex with men in 8 African countries: a cross-sectional analysis” |
|  |  | (*b*) Provide in the abstract an informative and balanced summary of what was done and what was found | Abstract, methods and findings section: “Data were merged and mixed effects logistic regression models were used to estimate relationships between gender identity and HIV-infection.” “Transgender women were more likely to report condomless sex during their most recent receptive anal sex act (OR = 2.13, CI: 1.79-2.50), to have been tested for HIV in the prior 12 months (OR = 1.28, CI: 1.08-1.51), and to be currently living with HIV (OR = 2.09, CI: 1.76-2.49). |
| Introduction | | |  |
| Background/rationale | 2 | Explain the scientific background and rationale for the investigation being reported | Introduction, paragraph 1: “Sub-Saharan Africa bears more than 70% of the global burden of HIV in the world….and no data were available from sub-Saharan Africa.” |
| Objectives | 3 | State specific objectives, including any prespecified hypotheses | Introduction, paragraph 4: “The objectives of this analysis were to estimate HIV prevalence among transgender women, characterize psychosocial and behavioral risk factors, and distinguish HIV epidemiology among transgender women from cis-MSM.” |
| Methods | | |  |
| Study design | 4 | Present key elements of study design early in the paper | Methods, paragraph 1: “secondary analysis of pooled data from multiple cross-sectional, bio-behavioral studies” |
| Setting | 5 | Describe the setting, locations, and relevant dates, including periods of recruitment, exposure, follow-up, and data collection | Methods, paragraph 2: “Data for these analyses were collected in urban settings as part of larger cross-sectional studies initially tailored for MSM. Data collection took place from 2011 to 2016 at 14 sites across eight countries: Bobo-Dioulasso and Ouagadougou in Burkina Faso (January – August 2013); Abidjan, Bouake, Gagnoa, and Yamoussoukro in Cote d’Ivoire (March 2015 – February 2016); Banjul in The Gambia (July – December 2011); Maputsoe and Maseru in Lesotho (February – September 2014); Lilongwe in Malawi (July 2011 – March 2012); Dakar in Senegal (February – November 2015); Mbabane in Swaziland (August – December 2011); and Kara and Lomé in Togo (January – June 2013).” |
| Participants | 6 | (*a*) *Cohort study*—Give the eligibility criteria, and the sources and methods of selection of participants. Describe methods of follow-up  *Case-control study*—Give the eligibility criteria, and the sources and methods of case ascertainment and control selection. Give the rationale for the choice of cases and controls  *Cross-sectional study*—Give the eligibility criteria, and the sources and methods of selection of participants | Methods, paragraph 3: “Participants were recruited using respondent driven sampling (RDS), except in the Gambia where a snowball sampling was used [18] . . . Eligible participants were age 18 years and older in all countries except the Gambia where enrollment included ages 16 years and older. Other eligibility criteria included being assigned male sex at birth and having had anal sex with a male partner in the prior 12 months.” |
|  |  | (*b*) *Cohort study*—For matched studies, give matching criteria and number of exposed and unexposed  *Case-control study*—For matched studies, give matching criteria and the number of controls per case | Not applicable |
| Variables | 7 | Clearly define all outcomes, exposures, predictors, potential confounders, and effect modifiers. Give diagnostic criteria, if applicable | Methods, paragraph 12:  Outcome 🡪 HIV test result  Exposure🡪 Transgender status  Predictors🡪 (a) Positive depression screen (b) condomless receptive anal sex (c) Experiences of stigma, including family stigma, law enforcement stigma, and violence.  Confounders🡪 Age  Effect modifiers🡪An interaction term for gender and condomless anal sex |
| Data sources/ measurement | 8* | For each variable of interest, give sources of data and details of methods of assessment (measurement). Describe comparability of assessment methods if there is more than one group | Methods, paragraphs 5-11:  Outcome 🡪 “A serial rapid HIV testing algorithm was implemented using Determine HIV-1/2® (Alere, Japan) for screening and Uni-GoldTM HIV (Trinity Biotech Ireland) for confirmation of positive results”  Exposure🡪 “transgender women were defined as participants who were assigned male sex at birth and self-identified as transgender or female/woman”  Predictors🡪 (a) “Depression screening was measured using an item that asked, “Have you felt sad or depressed in the last two weeks?” (b) “…they were asked if they had condomless insertive and/or receptive anal sex with any of their reported male partners.”  (c) “Experiences of stigma based on sexual orientation/practice were measured using 13 questions representing three primary forms of stigma including enacted, anticipated, and perceived.”  Confounders🡪 “Demographic data included age in years.”  Effect modifiers🡪“We hypothesized that gender would modify the relationship between condomless anal sex and HIV; therefore, an interaction term for gender and condomless anal sex was included in the final model.” |
| Bias | 9 | Describe any efforts to address potential sources of bias | Methods, paragraph 13: “The proportion of missing data was calculated for each variable in the model. Missing data comprised less than 5% of all variables except condomless anal intercourse which was missing for 14% of participant with no difference in proportion of missing data by gender. No data were imputed.” |
| Study size | 10 | Explain how the study size was arrived at | Methods, paragraph 1: “Pooled data from multiple cross-sectional, bio-behavioral studies.” |
| Quantitative variables | 11 | Explain how quantitative variables were handled in the analyses. If applicable, describe which groupings were chosen and why | Methods, paragraph 6: “Data sets from each country were merged and all questions that were in at least two surveys were kept for analysis. Consistent with global standards, transgender women were defined as participants who were assigned male sex at birth and self-identified as transgender or female/woman. Three countries (Burkina Faso, Lesotho, and Togo) also included the option to identify as intersex; however, all intersex participants also identified as either male/men or female/woman/transgender. Therefore, gender was dichotomized as transgender women and cisgender MSM.”  Also see Item 8 and Item 12. |
| Statistical methods | 12 | (*a*) Describe all statistical methods, including those used to control for confounding | Methods, paragraph 10: “Means and proportions were calculated for participant characteristics and variables of interest. . .Odds ratios were used to compare results between transgender women and cis-MSM using univariate logistic regression with a random intercept to account for clustering by site. A stigma index was created for item reduction using exploratory factor analysis (EFA) Mixed effect logistic regression models were built to estimate differences in HIV prevalence between transgender women and cis-MSM. A random intercept was added by site to account for clustering. Based on the primary objective to compare HIV prevalence |
|  |  | (*b*) Describe any methods used to examine subgroups and interactions | Methods, paragraph 12: “We hypothesized that gender would modify the relationship between condomless anal sex and HIV; therefore, an interaction term for gender and condomless anal sex was included in the final model.” |
|  |  | (*c*) Explain how missing data were addressed | Methods, paragraph 13: “The proportion of missing data was calculated for each variable in the model. Missing data comprised less than 5% of all variables except condomless anal intercourse which was missing for 14% of participant with no difference in proportion of missing data by gender. No data were imputed.” |
|  |  | (*d*) *Cohort study*—If applicable, explain how loss to follow-up was addressed  *Case-control study*—If applicable, explain how matching of cases and controls was addressed  *Cross-sectional study*—If applicable, describe analytical methods taking account of sampling strategy | Methods, paragraph 13: “Because we combined data from multiple studies, no adjustments were made for RDS sampling methods.” |
|  |  | (*e*) Describe any sensitivity analyses | Not applicable |

Continued on next page

| Results | | |  |
| --- | --- | --- | --- |
| Participants | 13* | (a) Report numbers of individuals at each stage of study—eg numbers potentially eligible, examined for eligibility, confirmed eligible, included in the study, completing follow-up, and analysed | In this cross-sectional, secondary analysis of existing data, all participants in each data set were included.  Results, paragraph 1: “… all participants were assigned male at birth, however 937 (20%) identified as transgender or female while 3,649 were cis-MSM.” |
|  |  | (b) Give reasons for non-participation at each stage | Not applicable |
|  |  | (c) Consider use of a flow diagram | Not applicable |
| Descriptive data | 14* | (a) Give characteristics of study participants (eg demographic, clinical, social) and information on exposures and potential confounders | Results, paragraph 1: Table 1. Participant Characteristics; Results, paragraph 3: Table 2. Psychosocial Factors among Transgender women and Cisgender MSM; Results, paragraph 4: Table 4. Sexual Risk and HIV/STIs among Transgender Women and Cisgender MSM. |
|  |  | (b) Indicate number of participants with missing data for each variable of interest | Methods, paragraph 13: “The proportion of missing data was calculated for each variable in the model. Missing data comprised less than 5% of all variables except condomless anal intercourse which was missing for 14% of participant with no difference in proportion of missing data by gender. No data were imputed.” |
|  |  | (c) *Cohort study*—Summarise follow-up time (eg, average and total amount) | Not applicable |
| Outcome data | 15* | *Cohort study*—Report numbers of outcome events or summary measures over time | Not applicable |
|  |  | *Case-control study—*Report numbers in each exposure category, or summary measures of exposure | Not applicable |
|  |  | *Cross-sectional study—*Report numbers of outcome events or summary measures | Results, paragraph 5: Table 5. HIV Prevalence by Country |
| Main results | 16 | (*a*) Give unadjusted estimates and, if applicable, confounder-adjusted estimates and their precision (eg, 95% confidence interval). Make clear which confounders were adjusted for and why they were included | Results, paragraph 5: Table 5. HIV Prevalence by Country [provides unadjusted odds ratio in final row of the table.] Page 17: Table 6. Multivariable logistic regression model of odds of HIV infection. |
|  |  | (*b*) Report category boundaries when continuous variables were categorized | Not applicable |
|  |  | (*c*) If relevant, consider translating estimates of relative risk into absolute risk for a meaningful time period | Not applicable |
| Other analyses | 17 | Report other analyses done—eg analyses of subgroups and interactions, and sensitivity analyses | Results, paragraph 6: Table 6. Multivariable logistic regression model of odds of HIV infection. |
| Discussion | | |  |
| Key results | 18 | Summarise key results with reference to study objectives | Discussion, paragraph 1: “In this analysis of data from MSM-tailored studies in eight countries across sub-Saharan Africa, one out of every five participants (n=937) identified as transgender and/or as a woman. Transgender women were more likely to report stigma, depressive symptoms, condomless receptive anal sex, and receipt of an HIV test within the prior 12 months compared with cis-MSM. In adjusted regression modeling, transgender women demonstrated a 2-fold higher odds of HIV infection than cis-MSM with significant effect modification of gender by condomless receptive anal intercourse.” |
| Limitations | 19 | Discuss limitations of the study, taking into account sources of potential bias or imprecision. Discuss both direction and magnitude of any potential bias | Discussion, paragraphs 8-9: “sampling strategies tailored for cis-MSM. . . cross-sectional nature, which limits causal inference. . . face-to-face interviews for data collection may have led to social desirability bias . . . potentially relevant variables were included if they only existed in one data set . . . interpretation of experiences of stigma was limited because some sites asked participants to attribute their experiences to sexual orientation and practices, while others did not.” |
| Interpretation | 20 | Give a cautious overall interpretation of results considering objectives, limitations, multiplicity of analyses, results from similar studies, and other relevant evidence | Discussion, paragraph 10: “Taken together, these data reinforce that gender identity is as complex in sub-Saharan Africa as in other regions, highlighting the need to collect and disaggregate data that distinguishes assigned sex at birth from current gender identity.” |
| Generalisability | 21 | Discuss the generalisability (external validity) of the study results | Discussion, paragraph 8: “However, the findings may not be generalizable to other transgender women in the region due to the use of sampling strategies tailored for cis-MSM.” |
| Other information | | |  |
| Funding | 22 | Give the source of funding and the role of the funders for the present study and, if applicable, for the original study on which the present article is based | Work in Togo and Burkina Faso was supported by Project SEARCH, which was funded by the US Agency for International Development under Contract GHH-I-00-07-00032-00 and by the President’s Emergency Plan for AIDS Relief (PEPFAR). Work in Côte d’Ivoire was funded by the Global Fund to Fight AIDS, Tuberculosis and Malaria through the Government of Côte d’Ivoire National AIDS Control Program (PNPEC) contract to Enda Santé, an organization based in Senegal, and subcontracted for technical assistance to Johns Hopkins University. Work in Lesotho was funded by the US Agency for International Development (USAID, AID-674-A-00-00001), and implemented by Population Services International/Lesotho (PSI). Work in Swaziland was funded by PEPFAR through the USAID Swaziland (GHH-I-00-07-00032-00). Finally, this publication was made possible with help from the Johns Hopkins University Center for AIDS Research, an NIH funded program (P30AI094189), which is supported by the following NIH Co-funding and Participating Institutes and Centers: National Institute of Allergy and Infectious Diseases (NIAID), National Cancer Institute (NCI), National Institute of Child Health and Human Development (NICHD), National Heart, Lung, and Blood Institute (NHLBI), National Institute on Drug Abuse (NIDA), National Institute of Mental Health (NIMH), National Institute on Aging (NIA), Fogarty International Center (FIC), National Institute of General Medical Sciences (NIGMS), National Institute of Diabetes and Digestive and Kidney Diseases (NIDDK), and the Office of AIDS Research (OAR). The content is solely the responsibility of the authors and does not necessarily represent the official views of the NIH. The funding sources had no role in the study design; in the collection, analysis and interpretation of data; in the writing of the articles; or in the decision to submit the manuscript for publication. |

*Give information separately for cases and controls in case-control studies and, if applicable, for exposed and unexposed groups in cohort and cross-sectional studies.

**Note:** An Explanation and Elaboration article discusses each checklist item and gives methodological background and published examples of transparent reporting. The STROBE checklist is best used in conjunction with this article (freely available on the Web sites of PLoS Medicine at http://www.plosmedicine.org/, Annals of Internal Medicine at http://www.annals.org/, and Epidemiology at http://www.epidem.com/). Information on the STROBE Initiative is available at www.strobe-statement.org.
